# Supplementary material for: Survival of COVID-19 with Multimorbidity Patients
Source: Healthcare (Basel). 2021 Oct 22;9(11):1423. doi: 10.3390/healthcare9111423 (PMC8617866; doi:10.3390/healthcare9111423)
Supplement: Supplementary file 1 [file healthcare-09-01423-s001.zip › healthcare-1364997-supplementary.pdf]

Supplementary Materials

Table S1. Sociodemographic and clinical characteristics of hospitalized cases for COVID-19 with multimorbidity in the state of Hidalgo in 2020-2021.

Characteristics	Censored		Failures	
	n	%	n	%
**Age**				
Under 65 years	4,961	77.7	3,057	54.9
Greater or equal 65 years	1,428	22.3	2,509	45.1
**Sex**				
Male	3,756	58.2	3, 619	65
Female	2,633	41.2	1,947	35
**Occupation**				
Unemployed	322	5.0	436	7.8
Employees	1,114	17.4	696	12.5
Students	54	0.9	8	0.1
Teachers	139	2.2	107	1.9
Others	794	12.4	618	11.1
Peasant/Driver/Worker	1,235	19.3	1033	18.6
Managers/other profesionals	217	3.0	139	2.5
Homme/Retiree/Pensioner	2,250	35.2	2424	43.6
Health Workers**	264	4.1	105	1.9
**Multimorbidity**				
No Multimorbidity	2240	35.1	1371	24.6
Diabetes/Hypertension	536	8.4	688	12.4
Hypertension	533	8.3	608	10.9
Obesity	675	10.6	397	7.1
Diabetes	599	9.4	479	8.6
Diabetes/Hypertension/Obesity	196	3.1	224	4
Hypertension/Obesity	209	3.3	209	3.8
Diabetes/Obesity	162	2.5	122	2.2
Diabetes/Hypertension/CKD	0	0.00	142	2.6
Hypertension/KCD	62	1.0	2	0.3
COPD	42	0.7	44	0.8
Other multimorbidity*	1135	17.8	1280	23.0
**Demand for medical attention**				
Less than 14 days	6,368	99.6	5,514	99.0
Greater or equal to 14 days	21	0.4	52	1.0

Use of mechanical ventilation				
No	6262	98.0	4186	75.2
Yes	127	2.0	1380	24.79

* Includes other multimorbidities such as: Immunosuppression, HIV, heart disease with other combinations

** Includes: Doctors, nurses, dentists, laboratory workers and other health workers. COPD: Chronic Obstructive Pulmonary Disease, CKD: Chronic Kidney Disease.

Table S2. Survival analysis after 40 days of hospital stay in hospitalized cases for COVID-19 with multimorbidity.

Characteristics	K-M Survival estimator %	*P* value associated with log-Rank Value
**Age**		
Under 65 years	19.2	<0.05
Greater or equal 65 years	7.00	
**Sex**		
Male	14.00	<0.05
Female	14.30	
**Occupation**		
Unemployed	14.30	<0.001
Employees	10.50	
Students		
Teachers	13.16	
Others	23.39	
Peasant/Driver/Worker	-	
Managers/other profesionals	19.05	
Home/Retiree/Pensioner	14.96	
Health Workers**	12.51	
**Multimorbidity**		
No Multimorbidity	14.8	<0.001
Diabetes/Hypertension	13.2	
Hypertension	11.3	
Obesity	24.0	
Diabetes	12.6	
Diabetes/Hypertension/Obesity	13.2	
Hypertension/Obesity	16.5	
Diabetes/Obesity	5.8	
Diabetes/Hypertension/CKD	6.3	
Hypertension/KCD	-	
COPD	-	
Other multimorbidity*	13.2	
**Demand for medical attention**		
Less than 14 days	14.30	<0.001
Greater or equal 14 days	10.50	
**Use of mechanical ventilation**		
No	24.50	<0.001
Yes	4.30	

* Includes other multimorbidities such as: Immunosuppression, HIV, heart disease with other combinations ** Includes: Doctors, nurses, dentists, laboratory workers and other health workers. COPD: Chronic Obstructive Pulmonary Disease, CKD: Chronic Kidney Disease.

Table S3. Prognostic factors associated with hospitalized cases for COVID-19 with multimorbidity in the state of Hidalgo in 2020-2021.

Characteristics	Risk Ratio<br>RR	95% IC
**Age**		
Under 65 years	1.00	
Greater or equal 65 years	1.53	1.44 - 1.63***
**Sex**		
Male	1.00	
Female	0.85	0.79 - 0.91***
**Occupation**		
Unemployed	1.00	
Employees	0.82	0.72 - 0.93***
Students	0.39	0.19 - 0.78***
Teachers	0.87	0.69 -1.09
Others	0.89	0.80 - 1.04
Peasant/Driver/Worker	0.88	0.79 - 1.00
Managers/other profesionals	0.73	0.62 - 0.92***
Homme/Retiree/Pensioner	1.01	0.90 - 1.13
Health Workers**	0.53	0.42 - 0.66***
**Multimorbidity**		
No Multimorbidity	1.00	
Diabetes/Hypertension	1.33	1.19 - 1.46***
Hypertension	1.13	1.02 - 1.24***
Obesity	1.02	0.94 - 1.14
Diabetes	1.14	1.03 - 1.27***
Diabetes/Hypertension/Obesity	1.32	1.19 - 1.54***
Hypertension/Obesity	1.21	1.04 - 1.41***
Diabetes/Obesity	1.15	0.95 - 1.40
Diabetes/Hypertension/CKD	1.77	1.47 - 2.13***
Hypertension/KCD	8.97	2.24 - 35.94***
COPD	1.14	0.83 - 1.55
Other multimorbidity*	1.26	1.16 - 1.37***
**Demand for medical attention**		
Less than 14 days	1.00	
Greater or equal 14 days	1.62	1.21 - 2.16***
**Use of mechanical ventilation**		
No	1.00	
Yes	1.45	1.36 – 1.54***

Regression Model of Proportional Risks. * Includes other multimorbidities such as: Immunosuppression, HIV, heart disease with other combinations ** Includes: Doctors, nurses, dentists, laboratory workers and other health workers. COPD: Chronic Obstructive Pulmonary Disease, CKD: Chronic Kidney Disease. *** P value <0.05

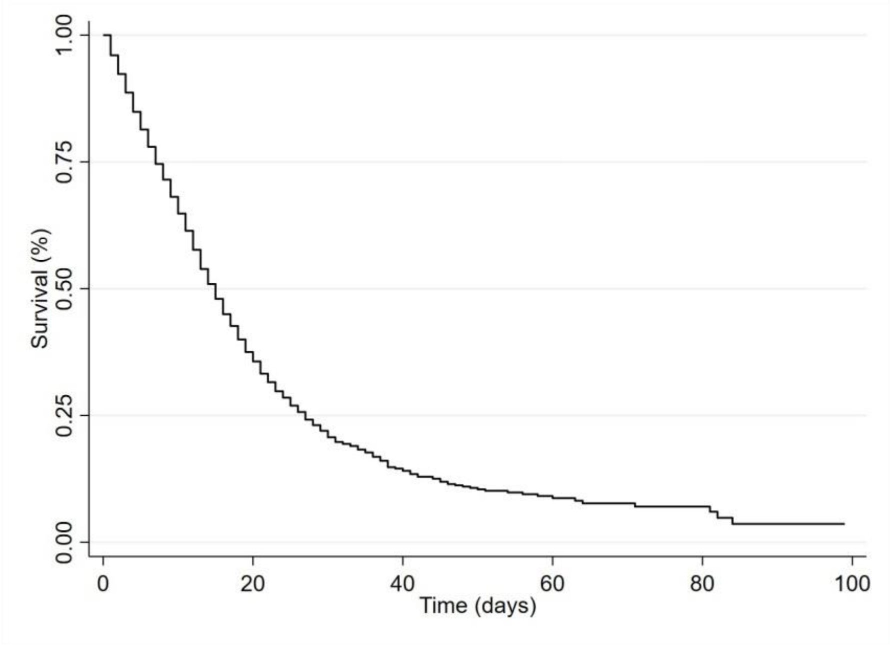

Figure S1. Kaplan-Meier estimator: Global Survival Curve of hospitalized cases for COVID-19 with multimorbidity in the state of Hidalgo in 2020-2021. The rate of survival of global population in days were recorded for each patient. Fifty percent decline occurred at 15 days, thereafter, a steady state curve appeared.
